# Sentiment analysis of popular-music references to automobiles, 1950s to 2010s

**DOI:** 10.1007/s11116-021-10189-1

**Published:** 2021-05-02

**Authors:** Chenyang Wu, Scott Le Vine, Elizabeth Bengel, Jason Czerwinski, John Polak

**Affiliations:** 1 School of Transportation, Southeast University, Nanjing, China; 2 Urban Systems Lab, Department of Civil and Environmental Engineering, Imperial College London, London, UK; 3 Transpo Group and Department of Geography, SUNY New Paltz, New Paltz, USA

**Keywords:** Peak car, Popular music, Sentiment analysis, Natural language processing

## Abstract

In recent years, there has been a scholarly debate regarding the decrease in automobile-related mobility indicators (car ownership, driving license holding, VMT, etc.). Broadly speaking, two theories have been put forward to explain this trend: (1) economic factors whose impacts are well-understood in principle, but whose occurrence among young adults as a demographic sub-group had been overlooked, and (2) less well-understood shifts in cultural mores, values and sentiment towards the automobile. This second theory is devilishly difficult to study, due primarily to limitations in standard data resources such as the National Household Travel Survey and international peer datasets. In this study we first compiled a database of lyrics to popular music songs from 1956 to 2015 (defined by inclusion in the annual “top 40”), and subsequently identified references to automobiles within this corpus. We then evaluated whether there is support for theory #2 above within popular music, by looking at changes from the 1950s to the 2010s. We demonstrate that the frequency of references to automobility tended for many years to increase over time, however there has more recently been a decline after the late 2000s (decade). In terms of the sentiment of popular music lyrics that reference automobiles, our results are mixed as to whether the references are becoming increasingly positive or negative (machine analysis suggests increasing negativity, while human analysis did not find a significant association), however a consistent observation is that sentiment of automobile references have over time become more positive relative to sentiment of song lyrics overall. We also show that sentiment towards automobile references differs systematically by genre, e.g. automobile references within ‘Rock’ lyrics are in general more negative than similar references to cars in other music genres). The data generated on this project have been archived and made available open access for use by future researchers; details are in the full paper.

## Introduction

There are two sharply distinct theories to explain the reversal of the long-term growth trends (a.k.a. ‘Peak Car’) in young people’s car-related mobility indicators (driving license holding, car ownership, driving mileage, etc.) observed in many high-income countries beginning in the 1990s/2000s (Blumenberg et al. 2016; Ciari and Axhausen 2015; Kuhnimhof et al. 2012). One school of thought focuses on changing economic circumstances and external constraints on young people’s mobility. This incorporates candidate explanators such as GDP per capita (Bastian et al. 2016), declining workforce participation and income levels (Bayart et al. 2020; Blumenberg et al. 2016; Delbosc and Currie 2014a), increasing costs of owning and operating a car (Bastian et al. 2016; Chatterjee et al. 2018; Klein and Smart 2017), and the advent of mechanisms that have made acquiring a driving license more onerous, time-consuming, and/or expensive (Thigpen and Handy 2018).

Such exogenous explanators by themselves have not been convincing to the research community as fully and completely accounting for young adults’ declining automobility indicators. Other factors such as possible shifts in cultural mores, values and sentiment among young adults towards the automobile have also been raised. McDonald, for instance, writes that “…there is not agreement among researchers…the second set of work [i.e. theory] acknowledges the importance of economic factors but argues that they do not fully explain observed declines…Identifying factors contributing to declines has been difficult, but [includes]…changing attitudes to travel generally and cars in particular. Some posit that the car is no longer a status symbol having been replaced by smartphones. Evaluating this is difficult” (McDonald 2017, pp. 3–5).

The difficulty in discriminating between these two competing schools of thought is at core due to data limitations: the data resources traditionally employed to both observe and model mobility trends (national and regional-scale household travel surveys) provide little or no information regarding respondents’ attitudes towards the car, and thus whether or not such attitudes may have shifted over time. Such datasets are relatively strong in demonstrating that young adults’ mobility indicators have shifted in unexpected ways since the 1990s/2000s [e.g. in the UK driving-license holding by people under age 30 peaked in the early 1990s, see Le Vine and Polak (2014)], however relatively weak in providing uncontested explanations for such trends. Although researchers have employed survey methods to probe attitude towards cars (Brown and Handy 2015; Thigpen and Handy 2018), the surveys are cross-sectional with limitations in capturing changing attitudes over time.

The motivation for the present research is thus to advance the state-of-knowledge regarding the hypothesis that attitudes towards the car have changed over time. Due to the data limitations noted in the previous paragraph, we devised a novel data-compilation strategy using lyrics from popular music covering a 60-year period from the 1950s through the 2010s. While such data cannot yield unambiguous conclusions about the possibility of social attitudes having shifted away from the car, this research strategy employs the corpus of popular music lyrics as an attempt to proxy for young adults’ social attitudes towards cars during this time period; we note that popular music is consumed most heavily by younger adults (Kalia 2015). Using the song lyrics, we then compiled a structured database of bars within the lyrics that reference automobility, and analyzed this database of ‘tokens’ using both sentiment analysis (a.k.a. “Natural Language Processing”) techniques and manual classification by the research team to quantitatively evaluate the research question.The datasets created for this paper have been archived at 10.17605/OSF.IO/UM5XB, and are available open access for future research use.

The remainder of this paper is organized as follows: “Literature review” section reviews the relevant literature, and Sect. “Data” section describes our data-compilation protocol. “Results” section then presents the results, and “Conclusions” section summarizes and concludes the paper.

## Literature review

This section first reviews the observations of declining automobile orientation among young adults and factors (both socio-economical factors and attitudinal factors) that appear relevant to the effect. We next review sentiment analysis approaches and their application in the context of transport, as well as the link between popular music and broader culture evolution.

### Young adults’ declining automobile orientation

Many researchers have investigated reasons for this effect over the past two decades, with a marked peak in research productivity on this line of enquiry in the period 2011–2015.

Table 1 summarizes factors that have been investigated and reported in the literature. Overall, socio-demographic characteristics and the built environment of their residence are the most extensively investigated factors. It appears to be broadly agreed that delayed transitions to adulthood (i.e. longer years of getting education and living with parents, delayed marriage), re-urbanization (i.e. living in dense urban areas with good public transport accessibility), and increased financial pressure on young people each play roles in the overall effect.

**Table 1:** Summary of literature in peak car

Socio-demographic features (i.e. income, gender, car access)	Built environment (i.e. population density, public transport access)	Values and attitudes (i.e. environment awareness, attitude towards cars)	ICT (i.e. smart phone and social media use)	Graduated Driving License
McDonald and Trowbridge (2009) Raimond and Milthorpe (2010) Williams (2011) Licaj et al. (2012) Kuhnimhof et al. (2012) Sivak and Schoettle (2012) Delbosc and Currie (2013) Delbosc and Currie (2014a) Delbosc and Currie (2014b) Le Vine et al. (2014b) Le Vine and Polak (2014) Schoettle and Sivak (2014) Tefft et al. (2014) Brown and Handy (2015) Ciari and Axhausen (2015) Curry et al. (2015) Baradaran et al. (2016) Hjorthol (2016) Delbosc and Nakanishi (2017) Thigpen and Handy (2018) Rérat (2018) Bayart et al. (2020) Vaca et al. (2020)	McDonald and Trowbridge (2009) Raimond and Milthorpe (2010) Williams (2011) Licaj et al. (2012) Sivak and Schoettle (2012) Delbosc and Currie (2013) Delbosc and Currie (2014b) Le Vine et al. (2014b) Le Vine and Polak (2014) Schoettle and Sivak (2014) Brown and Handy (2015) Baradaran et al. (2016) Hjorthol (2016) Thigpen and Handy (2018) Rérat (2018) Bayart et al. (2020) Vaca et al. (2020)	Williams (2011) Delbosc and Currie (2013) Delbosc and Currie (2014c) Le Vine et al. (2014a) Schoettle and Sivak (2014) Brown and Handy (2015) Fylan and Caveney (2018) Thigpen and Handy (2018)	Sivak and Schoettle (2012) Delbosc and Currie (2013) Delbosc and Currie (2014c) Delbosc and Currie (2014b) Le Vine et al. (2014b) Schoettle and Sivak (2014) Brown and Handy (2015) Thigpen and Handy (2018)	Raimond and Milthorpe (2010) Sivak and Schoettle (2011) Delbosc and Currie (2013) Tefft et al. (2014) Thigpen and Handy (2018)

The wide adoption of Information and Communication Technology (ICT) was expected by some to impact young people’s attitude towards cars. It was expected that the use of smartphones, social media and other electronic devices may decrease young people’s car ownership and car use, but results in the literature are mixed: Thigpen and Handy (2018) found that ICT is linked with delays in young people’s driving license acquisition, whereas Brown and Handy (2015) and Le Vine and Polak (2014) found the opposite. The authors are unaware of contributions to this research question published after the onset of the COVID-19 pandemic, during which ICT use has undergone a step change.

Graduated driving license (GDL) schemes have been implemented in many developed countries, such as Australia, Canada, New Zealand and US. Although implemented differently in different parts of the world, GDL usually comprises of three stages: learner permit, provisional license and full license. To pass through the stages, driving license applicants need to pass a series of tests, and fulfill requirements such as minimum age, minimum driving practicing hours, and/or minimum period of holding a learner permit/provisional license. Whether GDL helps explain reduced automobile orientation has been investigated on a number of studies, but the result again is mixed. Raimond and Milthorpe (2010) and Tefft et al. (2014) did not find evidence that GDL explains this effect, whereas Thigpen and Handy (2018) report that it does. It is worth noting that reduced automobile orientation among young adults is not homogenous across all social groups; for instance Williams (2011) reports that young people in the US that self-identify as not white are more likely to delay driving license acquisition than their white peers.

### Symbolic/affective motives for car use

Compared with other travel modes such as public transport, the private car appears to have greater psychological value attached (Jensen 1999). Beyond the car’s *instrumental* function (speed, flexibility, convenience, privacy, cargo-carrying, etc.), it has been argued that car users may also be motived by the *symbolic* (prestige, success, etc.) and *affective* (enjoyment of driving, feeling of control, independence, etc.) factors of cars (Steg 2005). Van and Fujii (2011) and Van et al. (2014) add an additional non-instrumental factor that they term *social orderliness* of travel modes, which captures environmental friendliness, safety, altruism, and quietness. Van and colleagues compared six Asian countries and report that in countries where intentions for car use are low, the non-instrumental factors are significant predictors of car commuting.

The symbolic/affective function of cars has been found to be associated with car ownership and driving license acquisition. For car ownership, Belgiawan et al. (2016) found that symbolic/affective attributes including ‘independence’ and ‘arrogant prestige’ have significant impact on Indonesian undergraduate students’ car purchasing behavior: those who think owning a car suggests independence are more likely to purchase a car, whereas those who think cars demonstrate arrogance are less likely to own one. Zhu et al. (2012) report similar results in their study among Chinese undergraduate students, in which the psychosocial valuations of cars dominate the aspiration for Chinese students’ car ownership in contrast to the instrumental valuations. For driving license acquisition, the literature contains studies that argue that attitudinal factors such as the intention of ‘being independent’ and ‘feeling driving to school is cool’ significantly stimulate young people to acquire a driving license (Fylan and Caveney 2018; Thigpen and Handy 2018).

It has been argued that the symbolic/affective function of cars may be less prominent in the youth of the Global North than in the lesser-developed Global South (Lyons 2015; McDonald 2015). A consistent pattern has also been reported within China, where the level of development in different regions is large. Zhu et al. (2012) found that students studying at a university in Zhenjiang (a third-tier city in China, less developed) value the non-instrumental values of cars much higher than their peers studying at Shanghai (a first-tier city, one of the most developed Chinese cities).

Given these reports of the possible impacts of attitudinal factors on car use, many of which have been collected in bespoke surveys at relatively small scale, documenting whether there has been a broad shift in attitudes towards cars is an important research question.

### Sentiment analysis

Sentiment analysis (SA), also termed opinion mining or emotion AI, aims at systematically identifying people’s opinions, attitudes and emotions towards an entity (Medhat et al. 2014; Pang and Lee 2008). These techniques are widely used in areas where the opinion of the customers/audiences is important. For example, SA has been used to address issues such as predicting election results (Choy et al. 2012; Ramteke et al. 2016), understanding customers’ view of products (Santhosh Kumar et al. 2017; Sari et al. 2018), and supporting investment decisions (Ren et al. 2019; Wu et al. 2014).

There are three main levels of classification in SA: document-level, sentence-level and aspect level (Liu 2012; Medhat et al. 2014). Document-level SA aims to identify whether an entire document presents a positive or negative sentiment. Sentence-level SA is more specific than the document level, but there is no clear and unambiguous threshold between document-level and sentence-level, as a sentence can be regarded as short document. Aspect-level SA is the most specific, which enables classification of the sentiment of aspects of phrases relative to a specific item or concept (termed the ‘entity’). For example, in the phrase *“the voice quality of this phone is not good, but the battery life is long”*, *“This phone”* is the entity, and *“voice quality”* and *“battery life”* are two aspects of the entity *“this phone”*.

SA techniques are subdivided into machine learning (ML) and lexicon-based approaches. The former relies on various ML techniques which include Naïve Bayes Classifier, Supportive Vector Machines Classifiers, Neural Networks, etc. The latter relies on a sentiment lexicon, a collection of known and precompiled sentiment terms. Many sentiment analysis algorithms have been proposed, and readers are referred to detailed reviews of sentiment analysis studies and algorithms (Mäntylä et al. 2018; Medhat et al. 2014; Yadav and Vishwakarma 2020).

Table 2 contains a summary of studies that have employed SA in the context of transport studies.

**Table 2:** Literature review of sentiment analysis in transport

Study	Objective	Data source	Geography	Algorithm/software used	Key findings
Qi et al. (2020)	A framework to extract and analyze public opinions on transport service	Twitter	Miami-Dade country, US	AFINN	After introducing sentiment variables, the prediction accuracy can rise 18.6% and reach ~ 90%
Kinra et al. (2020)	Public opinion about the adoption of autonomous vehicles	Twitter	Denmark	SentiStrengh	Text analytics can be used as a complement to surveys Safety, labor participation and congestion are the most important concerns
Mondschein et al. (2020)	Customer sentiment towards parking	Yelp	Phoenix, US	Lexicon-based algorithm	Sentiment about parking is in general negative Parking sentiment is part of the overall perception of customers toward a business Districts with more parking spaces per business tend to have more positive parking sentiment Parking is viewed more positively when shared parking facilities are provided
El-Diraby et al. (2019)	Customers’ satisfaction on transit service	Twitter	Vancouver, Canada	SentiStrength	Sentiment is in general negative Sentiments toward disruption, especially those related to public safety incidents, showed lower levels of negative sentiment The sentiment of the sub-network of the most influential players closely matched the topics and sentiment of the full network
Mendez et al. (2019)	An approach to capture user satisfaction with public transport	Twitter	Santiago, Chile	SentiStrength	The amount of bus stops and bus services covered by the proposed approach is larger than survey data The proposed approach is effective in diagnosing problems in a timely manner
Pratt et al. (2019)	Public opinion regarding ridesharing service	Twitter	US	Aylien	The number of negative tweets outweighs the number of positive ones about the service characteristics (like routing and travel time) Most tweets about other passengers feature “humor” about other passengers
Rahim Taleqani et al. (2019)	Public opinion regarding dockless bikesharing	Twitter	Multiple countries (primarily US)	Logistic regression, support vector machines, and naïve Bayes	The dockless bikesharing system receives more positive sentiments than negative ones The mostly mentioned sub-topics relevant to dockless bikesharing are electric scooters, private e-hailing companies, and blockage of sidewalks
Haghighi et al. (2018)	A framework to analyze public opinion on transit service quality	Twitter	Utah, US	Rsentiment proposed by (Bose et al. 2017)	The number of negative tweets is greater on weekends than weekdays Most negative tweets are related to transit routes with higher ridership There is potential to use social media data to analyze transit service quality
Kulkarni et al. (2018)	A system that can analyze public opinion on transport	Twitter	California, US	Valence Aware Dictionary and sEntiment Reasoner (VADER)	The quality of the system depends on the size of the dataset, the number of topics that are specific to the topic modelling algorithm, and the positive/negative thresholds of the sentiment analysis algorithm
Pai and Liu (2018)	Predict vehicle sales by sentiment analysis	Twitter	US	SentiStrength	Both social media sentiment and stock values have predictive power to forecast monthly total vehicle sales
Ali et al. (2017)	Using sentiment analysis to monitor transportation activities	Twitter	Not mentioned	SentiWordNet	The proposed approach can determine real-time traffic congestion mapping
Baj-Rogowska (2017)	Public opinion regarding Uber (ridehailing)	Facebook	Not mentioned	ProSuite	Sentiment analysis reflected events that affected the company’s reputation
Wijnhoven and Plant (2017)	Predict car sales by sentiment analysis	Coosto, Twitter, Facebook, LinkedIn, YouTube, Google + , Hyves, Instagram and Pinterest	The Netherlands	Coosto	Social media sentiments have little predictive power towards car sales, while Google Trends data and social mention volume have significant predictive power
Effendy et al. (2016)	Public opinion regarding public transport	Twitter	Indonesian	Support vector machines	The accuracy of sentiment analysis using support vector machine can reach 78%
Fraedrich and Lenz (2016)	Public interest in autonomous driving	Online comments on newspaper articles	Germany and US	Qualitative content analysis	Response to autonomous driving in different countries and different types of media is different Sentiment towards autonomous driving is generally positive, however the authors report finding some negative sentiments
Giancristofaro and Panangadan (2016)	Public opinion of the California Department of Transportation	Instagram	California, US	Support vector machines, naïve Bayes, and random forests	The precision of sentiment analysis can be improved if images and texts are combined
Hao et al. (2016)	Public opinion towards the I-710 Corridor Project	Twitter	California, US	API based on a naïve Bayes classifier	There are increasing twitter users participating the I-710 Corridor Project over time The number of comments from personal twitter accounts is positively correlated to the number of tweets from the organization account Twitter users are more likely to sent positive comments in the morning and negative comments in the afternoon towards I-710 Project Twitter users are more positive towards “Freeway Tunnel”, “Light Rail Transit” and improving the existing infrastructure, and more negative towards “Rapid Bus Transit”
Das et al. (2015)	Users’ sentiment toward bikesharing	Twitter	Washington DC, US	Lexicon-based algorithm	Most people view the bikesharing system positively
Luong and Houston (2015)	Public opinion on light rail service	Twitter	Los Angeles, US	Lexicon-based algorithm	Commuters mainly retweeted from other individuals or transit agencies, while schools and firms did not have strong retweet connections The Red Line was associated with the most positive tweets whereas the Blue Line has the most negative sentiments (of Los Angeles’ heavy rail lines)
Schweitzer (2014)	Public opinion regarding public transit	Twitter	US	Lexicon-based algorithm	Public transit receives the most negative comments Transit companies that respond to other social media users receive statistically more positive sentiments
Collins et al. (2013)	Transit riders’ satisfaction	Twitter	Chicago, US	SentiStrength	Riders express negative sentiments more than positive sentiments when an event occurs When an accident occurs, unusually high total social media engagement also occurs

Overall, the number of studies that employed SA in traveler attitude analysis has tended to increase. The objectives of these studies are frequently to understand public opinion on a specific mobility service, especially public transport and shared mobility services. Two of the studies analyzed the relationship between sentiments and car sales: Wijnhoven and Plant (2017) test the predictive power on car sales of the ratio of positive to negative tweets, the total number of mentions, the percentage of negative comments, and Google trends. Wijnhoven and Plant report that social media sentiments have relatively very weak salience to improve predictions of car sales. However, Pai and Liu (2018) conversely find that sentiment analysis of social media postings can improve the accuracy of regression models predicting monthly total vehicle sales in the US.

The authors are unaware of published literature that establishes how people’s attitudes towards private vehicles have evolved over the multi-decade period of interest (the latter part of the twentieth century and early part of the twenty-first century). Table 2 shows that the majority of studies have employed contemporary social media postings as the data source for SA, which do not provide information prior to the onset of social media in the 2010s. Hence, other data sources that provide an artefact of attitudes towards cars over a longer timescale are desirable.

### Cultural evolution in popular music

In general, popular music is more attractive to young people than other demographic groups (Kalia 2015). It has been argued that popular music helps youth to define their personal identity, serving to shape their behavior (Bogt et al. 2013) and partially reflecting matters that interest, worry, and concern its listeners (Christenson et al. 2019).

Similar to news media, the change of sentiment in popular music can potentially help document social changes. With respect to news media, Beckers et al. (2017) examined changing expectations for consumer price inflation in published news articles, and Cook et al. (2020) studied how references to drinking alcohol during pregnancy within newspapers have evolved over time. We are unaware of earlier literature discussing the association between popular music and young people’s attitude towards cars, but other aspects of cultural changes captured in popular music have been examined. For example, both Madanikia and Bartholomew (2014) and Christenson et al. (2019) find a significant increase (from the 1960s/70 s to the 2010s) in the proportion of songs with themes focusing on sex-related aspects of relationships, which likely reflects a cultural shift toward acceptance of sexuality outside of love relationships. Christenson et al. (2012) found an increase of songs referring to substance use, and in recent decades the use of alcohol and drugs were much likely to be portrayed positively.

In terms of the sentiment of music, popular music lyrics have in general tended to shift towards increasingly negative tone, from the 1950/60 s to the 2000s/10 s (Christenson et al. 2019; DeWall et al. 2011; Napier and Shamir 2018). Also, Pettijohn and Sacco found the sentiment of music to be linked with economic conditions. For music of the “Pop” genre, it is sadder, slower, and more comforting when the economy is experiencing hardship (Pettijohn and Sacco 2009). However, also during difficult economic periods, Country music has more positive lyrics than Pop, as well as being more musically upbeat and exhibiting the use of more happy-sounding major chords (Eastman and Pettijohn 2014).

This study addresses the gap by compiling a dataset that contains popular music lyrics over 60 years, with the objective of identifying whether there are changing patterns of references toward automobiles. We use both sentiment analysis algorithms and human analysts to identify the sentiment of songs and automobile references, and employ both descriptive analysis and regression to identify the association between sentiment towards cars and decades.

## Data

### Songs

The universe of popular music songs that we included in this analysis are the top 40 songs of each year in the US, as documented in the Billboard Year-End Hot 100 Singles (Billboard 2016), for the 60-year period 1956–2015.^1^ Lyrics for the songs in our sample were sourced from www.genius.com, and song genre information was sourced from www.iTunes.com. The datasets created for this paper have been archived at (Le Vine and Wu 2021), and are available open access for future research use.

**Fig. 1:**
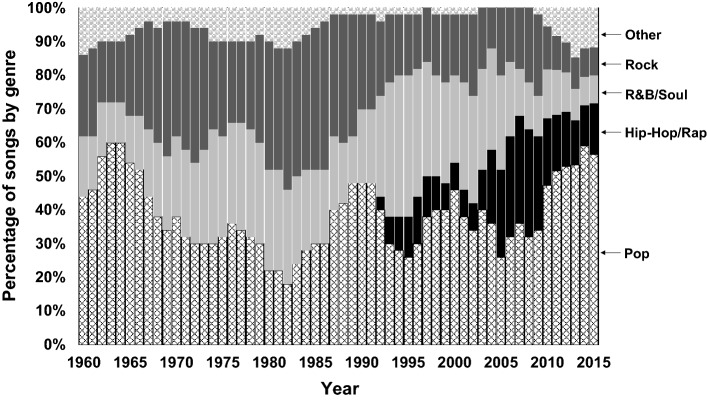
Percentage of music, by decade and genre

The distribution of songs by genre is presented in Fig. 1. The combined “Other” category of genres contains the following genres: Country, Easy listening, Electronic, Dance, Disco, Instrumental, Jazz, and Reggae.

It can be seen that Pop^2^ is generally the most common genre across time. R&B/Soul also tended to be a consistently common genre over time, but from the 1990s its share has decreased. The trend of the prevalence of Rock genre songs is similar to R&B/Soul. The number of Rock songs in the top 40 peaked around year 1980 and then decreased afterwards. On the other hand, Hip-Hop/Rap first appears in the early 1990s and has since increased rapidly to become the second most prevalent, though there has been a decrease since the late 2000s decade. This shift towards Hip-Hop/Rap has also been observed by others, e.g. Ryan (2018) and Guan (2017).

It has been reported that successful popular music songs in recent years have become more likely to be performed by female artists (Kaplan 2018). We found that this trend is significant for Pop music (*p* < 0.01) and for all genres combined (*p* < 0.01), but not for other genres besides Pop (*p* = 0.14).

### Automobile reference tokens

Upon compilation of the database of lyrics from the 2400 songs (60 years * 40 songs/year), we developed a set of uniform guidance for identifying tokens (and where to begin/end a token) within the lyrics that reference automobility (see “Appendix 1”). Two members of the study team then independently read the full set of lyrics to manually identify the tokens, yielding a token-identification match rate of 91%. Following a reconciliation process, the final database contains 535 tokens.

**Fig. 2:**
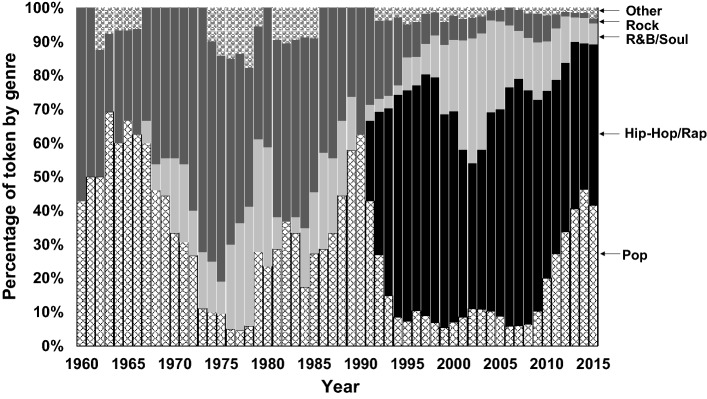
Of all references to cars, the percentage distribution by genre

The distribution of tokens by genre is presented in Fig. 2. It shows that, although Pop is the most common genre in most of the years (see Fig. 1), the frequency of automotive references in Pop music is relatively low, especially in the years around 1975 and 2000. Hip-Hop/Rap, on the other hand, shows a very high number of automotive references. Rock also has a high number of automobile references before the 1990s but has since decreased sharply. The frequency of automobile reference in different genres is discussed in more detail in the “Frequency of automobile references” section.

We next classified the tokens by various criteria, with the motivation to analyze how the types of references to cars have evolved over time. The eight criteria are:

- Cars (general)
- Car brands
- Car parts (see listing of observed car-part reference in “Appendix 1”)
- Car passenger travel
- Driving
- Stationary cars (as opposed to driving)
- Taxi/hitching a ride
- Traffic conditions

The eight criteria are not mutually exclusive. For example, *“We go to drive-in movies in a limousine/He takes me deep-sea fishing in a submarine”* was classified to belong to both *“Cars (general)”* and *“Driving”*. However, *“Cars (general)”* does not necessary include all other criteria. For example, *“Windshield wipers slapping time/I was holding Bobby's hand in mine”* was specified to belong to *“Car parts”* but not *“Cars (general)”*.

## Results

In this section, we first analyze general time trends in popular music (“Popular music trends, from 1950 to 2010s” section), followed by trends relating specifically to automobile references (“Frequency of automobile references” section) and then the sentiment towards cars (“Sentiment towards cars over time, Regression analysis” sections).

The change of popular music and the reference to automobile is analyzed by descriptive analysis, whereas the sentiment towards cars is also investigated by bivariate correlation and linear regression.

### Popular music trends, from 1950 to 2010s

**Fig. 3:**
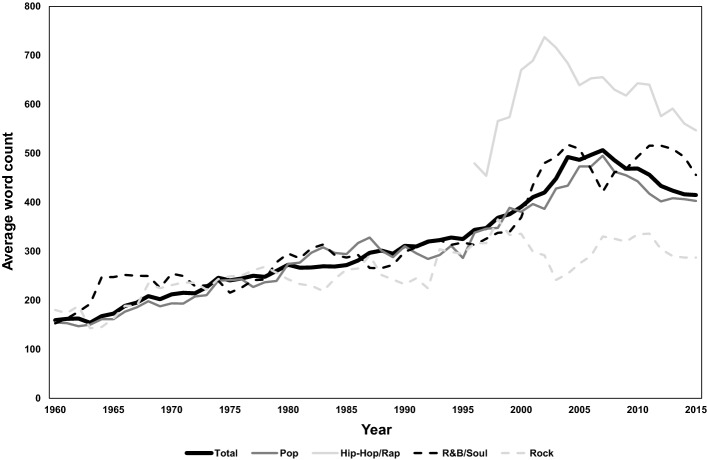
Average word count per song, by decade and genre

The change of average word count of the lyrics is presented in Fig. 3. We drop the category ‘Other’ from this point forward (as the number of songs belonging to this category is very small) and present only the four major genres.

The average word count has tended to increase over time, from under 200 words/song in the 1950s to 400–500 words/song in the 2000s/2010s. The average word count is much higher for Hip-Hop/Rap songs, followed by Pop and R&B, and lowest for Rock music. For Pop, R&B, and Hip-Hop/Rap, the average word count in general increases from the mid-1990s until year 2008 and drops afterwards. The time trend for Rock is different: word count has been more stable over time than for other genres. We note that the overall decreasing trend in words/song coincides with events that occurred in the late 2000s decade including the Global Financial Crisis, the rise of social media, and sustained increases in the price of gasoline; further investigation will be needed to establish the possibility of causality for any of these concurrent phenomena.

**Fig. 4:**
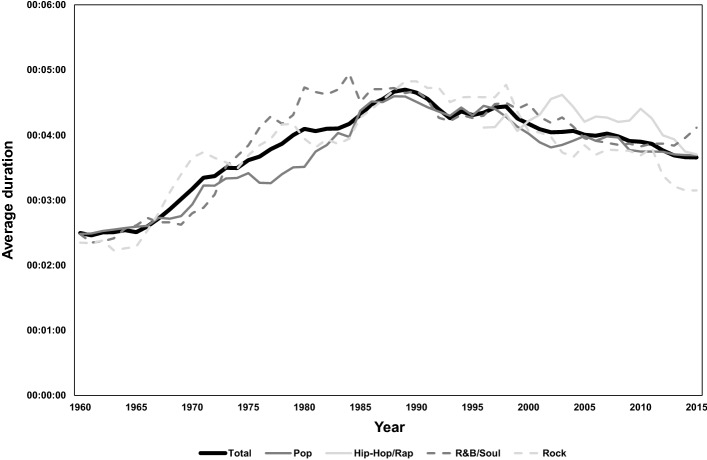
Average duration per song in minutes, by decade and genre

Figure 4 depicts the average duration of songs; this statistic peaked around 1990 and has subsequently dropped. Despite the higher average words/song of Hip-Hop/Rap, the duration of this genre is comparable to other genres. Overall, duration varies only weakly from genre to genre. Thus Hip-Hop/Rap songs tend to be characterized by much higher words/minute than other genres (i.e. 147 words/min in 2011–2015, compared to 107 words/min for all other genres combined during this period).

### Frequency of automobile references

**Fig. 5:**
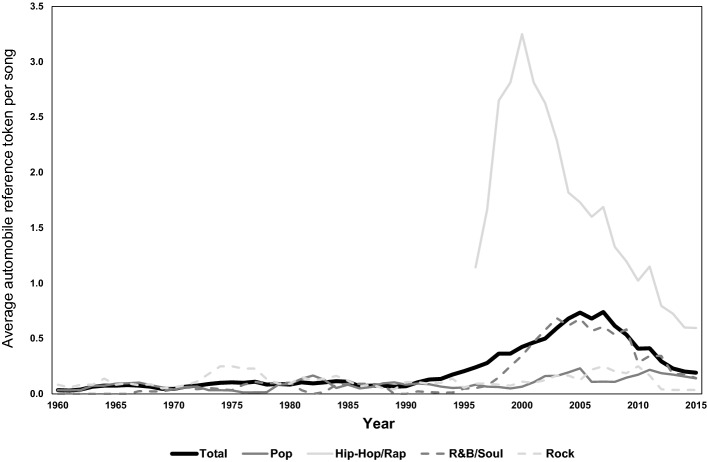
Average number of automobile references per song

Figure 5 shows that the number of automobile references per song peaks at around year 2005 and drops afterwards. The change of automobile references over time is similar to the time-trend of word count (see Fig. 3): The number of automobile references per song was low and stable until the 1990s, then tended to increase until the late 2000s decade, and has since experienced a decreasing trend. Even with this post-2008 decreasing trend, the frequency of automobile references in the 2010s is high compared to the 1950s–1990s period. Again, our dataset does not indicate the reason(s) behind these patterns, thus we must leave them as items for future investigation.

It is noteworthy that this ‘peak’ in automobile references in popular music coincides very roughly with the ‘peak’ in car orientation among young US adults, possibly lagging it by several years. On the latter of these points, Kuhnimhof et al. (2012) report that car mileage per US adult age 20–29 decreased approximately 20% between the 2001 and 2008 waves of the National Household Travel Survey (NHTS). NHTS data were not collected for any years between 2001 and 2008, thus the time-trend in this statistic within this period is not knowable. However, Kuhnimhof et al. (2012) document that across five other high-income countries that have historical national travel survey datasets collected at differing frequencies and in different years, the ‘peak’ in this statistic also appears to have occurred *“around the turn of the millennium”* (p. 772). In terms of license-holding, Delbosc (2017) finds that youth licensing in the US declined from a ‘peak’ in the late 1990s (i.e. near the turn of the millennium, but clearly prior to the 2008 ‘peak’ in car references in popular music), mainly due to subsequent decreases in license-holding by teens, with the license-holding rates of young adults in their mid-20 s remaining more stable.

The increase in car references beginning around 1990 coincides with the increasing prevalence of Hip-Hop/Rap in the top 40, and automobiles are referenced at a much higher frequency in Hip-Hop/Rap songs than other genres, especially in the years leading up to the turn of the millennium (see Fig. 5). In the post-2000 period, car references in Hip-Hop/Rap have decreased sharply, however remain much higher than other genres. Hence, the popularity of Hip-Hop/Rap music is a partial explanation for the higher frequency of automobile references in more recent decades (if the genre mix in 2015 were the same as the year 1990, the number of automobile references would have been only 0.07/song, compared to the actual observation of 0.19/song).

**Fig. 6:**
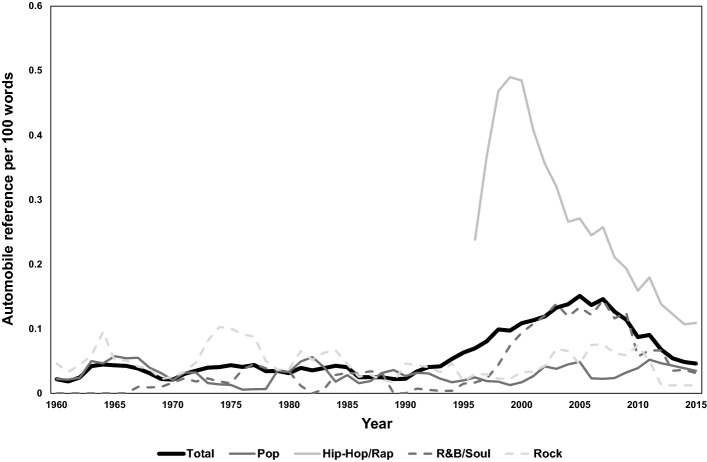
Average number of automobile references per 100 words

To disentangle between the trend of word count shifting over time simultaneously with the changing frequency of car references, we examined the average number of automobile references per 100 words (see Fig. 6).

We find that the trend of curves in Fig. 5 (car references per song) and Fig. 6 (car references per 100 words) are in general similar; it can therefore be concluded that the changing words/song is not a satisfactory explanation for the change over time in the number of automobile references.

**Fig. 7:**
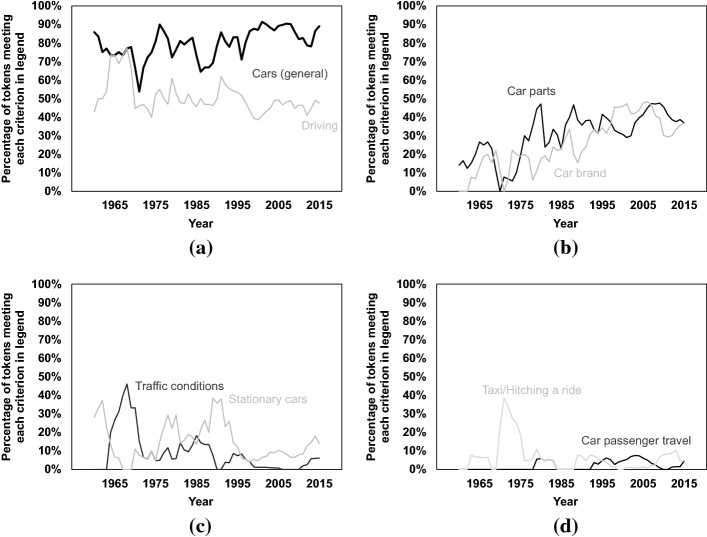
Percentage of tokens meeting various criteria (see label of each curve)

Figure 7 shows the change over time in the proportion of car references meeting each of the eight criteria listed in “Automobile reference tokens” section. We group the eight criteria into four groups:

- Fig. 7 panel (a) shows the change of tokens associated with *cars (general)* and *driving*. In general, tokens associated with these two criteria are consistently high in all these years, and there are no major time trends.
- Fig. 7 panel (b) shows the change of tokens associated with *car parts* and *car brand*. There is an increasing trend over time in the frequency of tokens associated with these two criteria.
- Fig. 7 panel (c) shows the change of tokens associated with *traffic conditions* and *stationary cars*. They are mentioned at a lower frequency compared to Fig. 7 panels (a) and (b), and there are no major time trends for these two criteria.
- Fig. 7 panel (d) shows the change of tokens associated with *taxi/hitching a ride* and *car passenger travel*. They are mentioned at the lowest frequency among all groups, and no clear trend over time is observed.

**Fig. 8:**
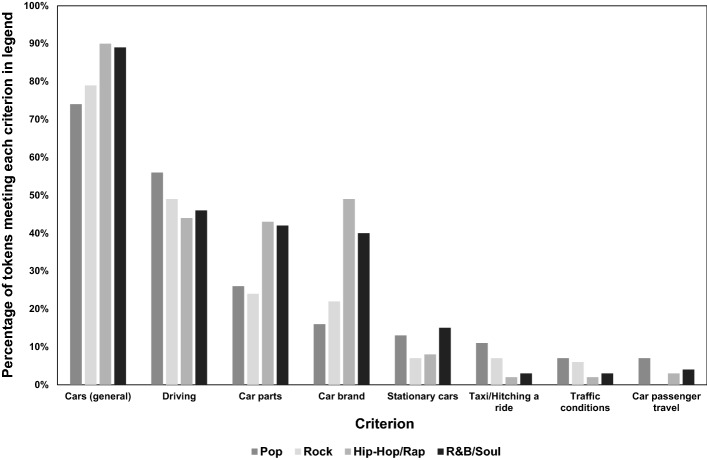
Percentage of tokens meeting each criterion in legend, by genre

We then investigate the relationships between genre and each of these eight criteria; Fig. 8 contains their cross-tabulation.

We can see that *Cars (general)* and *Driving*, which are the most frequently mentioned criteria, are mentioned at a similar frequency across the four music genres. In contrast, *Car parts* and *Car brands* are mentioned more frequently in Hip-Hop/Rap and R&B/Soul music than in other genres. The number of tokens matching the other four criteria (i.e. at the right hand side of Fig. 8) is very low, hence meaningful comparisons cannot be drawn.

### Sentiment towards cars over time

To perform the Sentiment Analysis, we employ two open-source algorithms: IBM “Alchemy Language” (which at the time of writing has been integrated into the Watson line of products)^3^ and IBM Watson “Tone Analyzer.^4^

Both algorithms use Machine Learning approaches to identify sentiments, and have been widely applied to text sources including customer reviews (Gao et al. 2015; Shah et al. 2020) and social media posts (Cao et al. 2018; Jussila and Madhala 2019).

Their application in Sentiment Analysis of popular music lyrics is relatively rare. We are aware of two examples: Al Marouf et al. (2019) investigated the use of IBM Watson Tone Analyzer to analyze language and emotional tones in lyrics; and Napier and Shamir (2018) analyzed 6150 Billboard 100 songs from 1951 to 2016, reporting that popular music is tending over time to exhibit increasingly negative sentiment.

The ‘Alchemy Language’ algorithm yields output of ‘positivity/negativity’ of the textual input’s sentiment on a continuous scale of − 1.0 (strongly negative sentiment) to + 1.0 (strongly positive sentiment).

The ‘Tone Analyzer’ algorithm’s output includes scores of ‘emotion’ on a continuous scale of 0.0–1.0, for five emotions: Anger, Disgust, Fear, Joy, and Sadness. For the purposes of this research, we employ only the ‘Joy’ emotion score, and “Joy Watson” is used from here onwards to refer to this algorithm. Joy is defined for use in the algorithm as: *Joy or happiness has shades of enjoyment, satisfaction, and pleasure. There is a sense of well-being, inner peace, love, safety, and contentment* (Mahmud 2016).

In addition to the objective outputs provided by the two algorithms, two members of the research team also independently manually classified each ‘token’ (reference to automobility) on a binary scale (− 1 for negative, + 1 for positive). Table 3 shows the correlation matrix between the scores from the two algorithms and the two members of the study team. It can be seen that the correlations are much stronger between the outputs of the two algorithms (0.44) and between the outputs of the two human analysts (0.60) than between the algorithms and human analysts (all between 0.09 and 0.16; all are statistically significant at *p* < 0.05).

**Table 3:** Correlation matrix comparing coding outputs for car token sentiments

	Alchemy	Joy Watson	Human analyst 1^a^	Human analyst 2
Alchemy	1.0	0.44	0.16	0.13
Joy Watson		1.0	0.13	0.09
Human analyst 1			1.0	0.60
Human analyst 2				1.0

^a^See acknowledgments for identification of human analysts

**Fig. 9:**
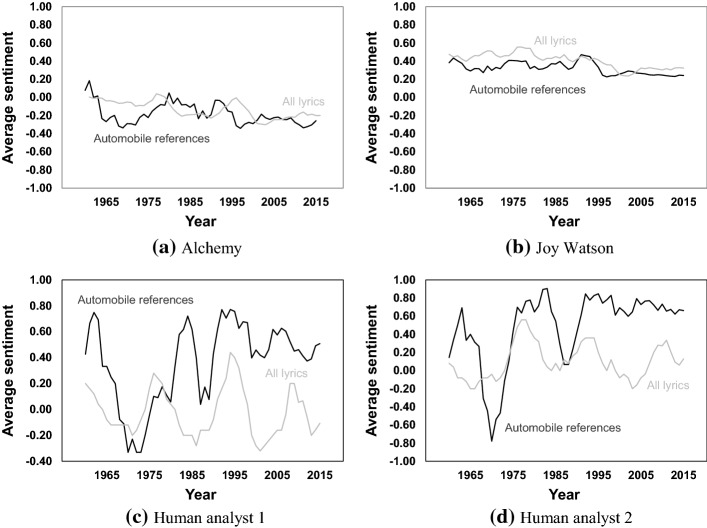
Average sentiment of songs and tokens

We first document the change in sentiment towards cars over time, as shown in Fig. 9.^5^ To determine whether this trend is independent of the concurrent trend in overall sentiment of all-lyrics (i.e. a background trend), the latter is also presented in Fig. 9.

Unlike the frequency of automobile references, there is not a clear trend break in sentiment of them (or of all lyrics) in the post-2000 time period.

It can also be seen that the results from the algorithms and human analysts are quite different. The algorithms show a decreasing trend in the sentiment of both automobile references and all popular music lyrics over time. However, the human analysts’ evaluations do not show a clear time trend for all-lyrics, and show an increasing trend in sentiment of references to automobiles (these time trends are confirmed in the correlation analysis presented below in Table 4).

**Table 4:** Correlation between sentiment and year (bold indicates significant at *p* < 0.05)

	Alchemy		Joy Watson		Human analyst 1		Human analyst 2	
	Automobile references	All lyrics	Automobile references	All lyrics	Automobile references	All lyrics	Automobile references	All lyrics
Pearson’s correlation between sentiment and year	**− 0.09** (*p* < 0.01)	**− 0.20** (*p* < 0.01)	**− 0.16** (*p* < 0.01)	**− 0.22** (*p* < 0.01)	**0.12** (*p* < 0.01)	**− **0.04 (*p* = 0.28)	**0.17** (*p* < 0.01)	0.03 (*p* = 0.46)

A possible reason for the humans-algorithms differences is that the two algorithms are not trained specifically by music lyrics. The algorithm designers do not disclose the types of datasets used to train the two algorithms, however it is known that applications of the two algorithms have included social media postings and hotel reviews (Cao et al. 2018; Gao et al. 2015; IBM 2019). Of the two studies of which we are aware that employ the *Watson Tone Analyzer* algorithm on music lyrics (Al Marouf et al. 2019; Napier and Shamir 2018), both used only the algorithm’s determinations, without the inclusion of a comparison against human analysts’ judgments.

Comparing song lyrics to hotel reviews and social media, the syntax and content is quite different, for various reasons (choice of words constrained by need to rhyme, lack of sentence structure, use of double-entendres, audio cues such as voice tone that carry meaning but have no analogue in written text, etc.). Such differences may explain part or all of the divergence between the sentiment assigned by the human analysts and by the algorithms.^6^

A bivariate correlation (presented in Table 4) was undertaken to test whether the correlation between sentiment and year is significant. For sentiment scores obtained from the two algorithms, there is negative and significant association between sentiment and year, for both automobile references and all-lyrics, but the former is less negative. For the two human analysts, the association between automobile-reference sentiment and year is positive and significant, there is no significant association between all-lyrics sentiment and year, and the correlation between automobile references and year are also more positive than the same for all-lyrics.

Thus, while the humans’ analyses and sentiment algorithms’ analyses differ in the absolute correlation with time, they concur that automobile references have become more positive relative to all-lyrics.

**Fig. 10:**
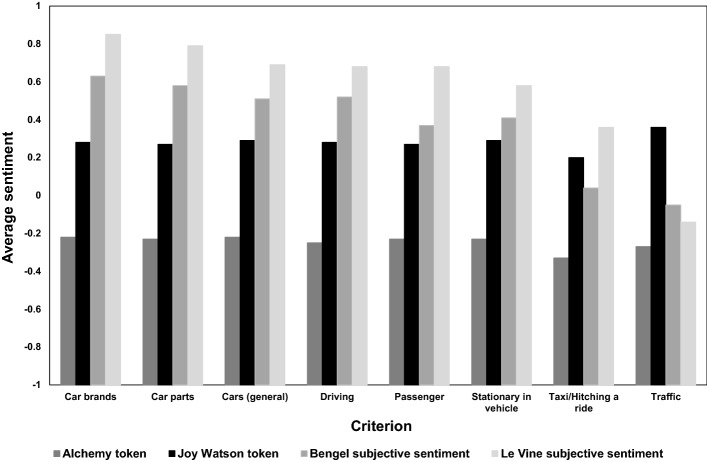
Average sentiment of tokens meeting each criterion in legend

Figure 10 presents analysis of the sentiment of automobile references, by both the algorithms and human analysts. The two human analysts found references to Car Brands and Car Parts to be the most positive, and references to Taxis/Hitching a ride and Traffic conditions to be the most negative. The two algorithms, by contrast, show little variation in positivity/negativity of sentiment with respect to the eight criteria.

### Regression analysis

The cross-tabulation results presented in “Popular music trends, from 1950 to 2010s”–“Sentiment towards cars over time” sections demonstrate that there is systematic variation in references to automobiles, with respect to year, genre, automobile reference criteria, etc. In this section, we present results of regression analysis to estimate the strength/sign of the associations, and establish which are all else equal when considered simultaneously.

The independent variables included in the specification are:

- year each song was published,
- the gender of the artist (1 = female, or the percentage of members that are female in the case of multi-member group artists),
- the classification of the token (as presented in “Automobile reference tokens” section), and
- the genre of the songs as independent variables.

The sentiment scores of the tokens and songs that are obtained from the two algorithms (Alchemy and Joy Watson) and the two human analysts serve as dependent variables.

We first present, inTable 5,^7^ the regression results of sentiment of tokens vs independent variables. Overall, all four models are statistically significant, but the goodness-of-fit of the two algorithm models are particularly low. Similar to results presented in Table 3, results from the two algorithms show similar patterns, as do the results from the two human analysts. However, results from algorithms and human analysts differ more sharply.

**Table 5:** Regression analysis for sentiment of automobile references

	Alchemy		Joy Watson		Human analyst 1		Human analyst 2	
	Coefficients	*p *value	Coefficients	*p *value	Coefficients	*p *value	Coefficients	*p *value
Constant	7.92	0.01	6.42	< 0.01	0.55	< 0.01	0.43	< 0.01
Year	**− **0.04	< 0.01	**− **0.03	< 0.01	*		*	
Percentage of female artists	*		*		**− **0.31	< 0.01	*	
Genre: Pop	0 (fixed)		0 (fixed)		0 (fixed)		0 (fixed)	
Genre: Hip-Hop/Rap	*		**− **0.05	0.04	*		0.29	< 0.01
Genre: R&B/Soul	*		*		**− **0.23	0.02	*	
Genre: Rock	**− **0.09	0.13	**− **0.07	0.04	**− **0.57	< 0.01	**− **0.28	< 0.01
Genre: Country	*		*		*		*	
Genre: Other	*		*		*		*	
Criteria: Cars	*		*		*		*	
Criteria: Traffic	*		*		**− **0.49	< 0.01	**− **0.70	< 0.01
Criteria: Driving	*		*		0.14	0.07	0.09	0.13
Criteria: Passenger	*		*		*		*	
Criteria: Stationary in Vehicle	*		*		*		*	
Criteria: Car Parts	*		*		*		0.12	0.06
Criteria: Brand	*		*		0.21	< 0.01	0.19	< 0.01
Criteria: Taxi/Hitching a Ride	*		**− **0.11	0.02	*		*	
Adjusted $r^{2}$	0.01		0.05		0.10		0.15	
Model significance	0.03		< 0.01		< 0.01		< 0.01	

^*^Factors with *p *value > 0.15 were excluded

As shown in Table 5, even when the influence of the independent variables is taken into account (genre, gender of artists, etc.), the negative association between sentiment of tokens and year remains significant for the two algorithm models. For the two human analysts’ models, the association becomes insignificant.

We hypothesized that artist gender may be associated with sentiment of automobile references, as discussed in “Songs” section. Results on this point are mixed: the all else equal effect of a song being performed by a female artist(s) was negative and significant in one human-analyst model, and not significant in the other three models.

For both algorithm and human analysts’ models, the genre Rock is significantly and negatively associated with sentiment of automobile references. The effect of Hip-Hop/Rap genre is found to be negatively associated with the sentiment for the “Joy Watson” model (*p* = 0.04). However, the Human Analyst #2 model finds a significant and positive association between the sentiment of automobile references and Hip-Hop/Rap genre (*p* < 0.01).

In terms of the eight criteria describes in “Automobile reference tokens” section, the majority of effects are insignificant. Two noteworthy findings are:

1. Both algorithms find an all else equal negative link between the *Taxi/Hitching a ride* category and sentiment, and
2. Both human analysts find positive all-else-equal relationships between sentiment and *Car Brand* (*p* < 0.01 for both), and negative association between sentiment and *Traffic* (*p* < 0.01 for both). A positive but weaker relationships is found between sentiment and *Driving* (*p* = 0.07; *p* = 0.13)

In summary, the clearest and most consistent finding from the regression analysis, which holds across both humans and both algorithms, is the negative all-else-equal effect of a song belonging to the Rock genre. Interestingly, in regression analysis with sentiment of all-lyrics as the dependent variables (and otherwise analogous to the regression analysis presented in this section, see “Appendix 2”), we also found across all humans and algorithms that Rock genre is negatively associated with all-song sentiment.

Finally, beyond this consistent observation with respect to Rock genre, we also found several other relationships that held across either humans or algorithms, but not across both of them.

## Conclusions

In this study we first developed a novel database of references to automobility in popular music in the period 1956 to 2015, and subsequently interrogated this database to determine whether there have been systematic shifts in frequency of references and/or sentiment to automobiles over time. The lyrics of popular music songs is an ideal corpus for this analysis as it is continuously available over many decades, freely available to researchers, and is a historical artefact of data on attitudes that could not readily be compiled in the present day by survey methods. Several background trends in popular music (time trends in word count per song, song duration, all-lyrics sentiment, etc.) are observable; we undertook efforts to disentangle between these background trends and effects related specifically to automobile references within the lyrics.

On the motivating research question—whether there is empirical support for the “changing attitudes towards cars” hypothesis to explain the decline in young adults’ car-borne mobility—our conclusions are mixed; they diverge in terms of frequency-of-car-references and their sentiment. Specifically, our main findings are:

1. A general upwards trend over time in the frequency of references to cars until the late 2000s, and a downward trend since (but remaining historically high). This inflection point coincides very roughly with findings by others that car mileage (Kuhnimhof et al. 2012) and driving license-acquisition (Delbosc 2017) in high-income countries began to decline “*around the turn of the millennium*” (Kuhnimhof et al. 2012, p. 772).
2. Mixed results as to whether sentiment of these references has become more positive or more negative over time. Human-classification suggests increasingly positive sentiment of references to automobiles, however the sentiment analysis algorithms indicate the opposite. Unlike point #1 above, we did not find a clear trend break in sentiment to automobiles (within popular music lyrics) in the post-2000 period.
3. Although the trends found by humans and algorithms are different, a consistent observation is that sentiment of automobile references have over time become more positive relative to sentiment of song lyrics overall.

We also report a minor finding: for both automobile references and all lyrics, the genre Rock is negatively associated with sentiment (across both human and algorithm analysis).

The datasets created for this paper have been archived at (Le Vine and Wu 2021), and are available open access for future research use.

We now conclude with a brief discussion of future research needs to advance this line of enquiry. First, the divergence of results between the sentiment analysis algorithms and human analysts needs more investigation; it may relate in part to the types of datasets used to train the algorithms, which are likely to be quite different from the non-traditional syntax and content that characterizes song lyrics. Second, other historical artefacts of late-20th/early-twenty-first century culture (e.g. newspaper/magazine archives, movie/television scripts, etc.) would be very useful, to enrich the findings we present and identify the extent to which they support the results from popular music lyrics. A promising direction would be to examine corpuses of text targeted at different demographic segments (as people belonging to different demographic group prefers different media), in recognition that the ‘Peak Car’ effects vary across demographic groups. An important direction for future research would be to establish whether the findings we present could be applied to influence attitudes as form of transport policy intervention.

Third, the fact that several indicators within popular-music lyrics appear to have trend breaks around late 2000s suggests that researchers should focus attention on this period. Fourth, international comparison across different societies, beyond the US, would also be potentially powerful, including both highly motorized societies (e.g. Germany, Japan) and those in the earlier stages of motorization (China, India, Brazil, etc.)

In closing, it is hoped that this line of enquiry will help the research community to distinguish between the ‘economic’ and ‘attitudinal’ theories to explain the decline in young adults’ automobility.

## Appendix 1: Rules employed during token-classification, listing of excluded ambiguous references, and listing of car parts

### General rules of token-identification

1. Reference must be to cars, traffic (flow of multiple vehicles), driving/passenger travel, car parts, or taxi/hitching a ride. Not roads. Not motorcycles. Yes trucks/RVs/ambulances/police-cars. Not when car terms are used purely allegorically (e.g. ‘junk in the trunk’ to refer to body shape)
2. To identify each token: Line referencing “cars”, plus the previous line and/or following line if each rhymes. If two tokens defined this way are directly adjacent to one another, we define it as one token.
3. We remove non-word noises (oop, oop, oop; dum-dee-dum, oh, etc. from the middle of tokens), to avoid confusing the algorithms
4. We change spellings in tokens to standard spellings, and clean up syntax (e.g. “I’ma” to “I’m going to”)
5. We remove racial/ethnic slurs from lyrics before processing them through the algorithms.

### Specific borderline (ambiguous) references that were explicitly excluded:

Lover’s lane; Anything to do with a mechanic or garage or chop shop or gas station or car wash or drive-in unless token mentions cars as vehicles or driving; Hit the road; Roll with me; Buses; Motor City; Dead Man’s Curve; Took a wrong turn; Ride up; Go-karts; Bumper cars; Toot-toot, beep-beep; Call me for a ride; Let’s cruise, away from here; Motoring; Let me start you up; Pull up; Living in the fast lane (but yes to “speeding in the fast lane”); Buckle up.

### List of specific cars parts that are referenced at least once

Radio, keys, brake, engine, turbo (charger), motor, engine noise (purr), window, glass, motor, white wall tires, sirens, roof, bumper, door, stereo, top, buckle, seat belt, honk, steering wheel, spare tire, trunk, amps, (gas) tank, hydraulics, front, back, passenger side, rear view, 18′s (rims), gas, fuel, wood panel, chrome, speaker/subwoofer, sun roof, title, ashtray, lift gate, throttle, license plate, ignition, paint color, paint type (e.g. candy gloss), TVs, car phone (car celly), rag top, chrome pipes, chrome hydraulics, low pro (file tires), car seats, stainless wheels, chrome wheels, white leather inside, alarm, bucket seat, butterfly doors, pedal, gears, dash, AC, button.

## Appendix 2: Regression analysis of all-lyrics sentiment (see discussion in “Regression analysis” section)

See Table

**Table 6:** Regression analysis for sentiment of songs (i.e. all-lyrics)

	Alchemy		Joy Watson		Human analyst 1		Human analyst 2	
	Coefficients	*p *value	Coefficients	*p *value	Coefficients	*p *value	Coefficients	*p *value
Constant	7.98	< 0.01	8.15	< 0.01	8.07	0.08	0.16	< 0.01
Year	$- 4.09 \times 10^{ - 2}$	< 0.01	$- 3.89 \times 10^{ - 2}$	< 0.01	$- 4.09 \times 10^{ - 2}$	< 0.01	*	
Percentage of female artists	*		*		*		*	
Automobile reference	*		*		*		*	
Genre: Pop								
Genre: Hip-Hop/Rap	*		*		0.33	0.04	0.28	0.06
Genre: R&B/Soul	0.08	0.01	*		0.27	< 0.01	*	
Genre: Rock	**− **0.09	0.04	**− **0.06	0.05	**− **0.18	0.07	**− **0.33	< 0.01
Genre: Country	*		*		*		*	
Genre: Other	0.22	0.11	*		*		*	
Adjusted $r^{2}$	0.08		0.05		0.03		0.03	
Model significance	< 0.01		< 0.01		< 0.01		< 0.01	

^*^ Factors with *p *value > 0.15 were excluded

6.

## Appendix 3: Regression analysis of token sentiment (10-year band model and before-after peak car model)

See Tables 7 and 8.

**Table 7:** Regression analysis for sentiment of automobile references (10-year band model)

	Alchemy		Joy Watson		Human analyst 1		Human analyst 2	
	Coefficients	*p *value	Coefficients	*p *value	Coefficients	*p *value	Coefficients	*p *value
Constant	0.14	0.11	0.45	< 0.01	0.55	< 0.01	0.43	< 0.01
Decade	**− **0.05	< 0.01	**− **0.03	< 0.01	*		*	
Percentage of female artists	*		*		**− **0.31	< 0.01	*	
Genre: Pop	0 (fixed)		0 (fixed)		0 (fixed)		0 (fixed)	
Genre: Hip-Hop/Rap	**− **0.08	0.15	**− **0.05	0.03	*		0.29	< 0.01
Genre: R&B/Soul	*		*		**− **0.23	0.02	*	
Genre: Rock	**− **0.17	< 0.01	**− **0.06	0.06	**− **0.57	< 0.01	**− **0.28	< 0.01
Genre: Country	*		*		*		*	
Genre: Other	*		*		*		*	
Criteria: Cars	*		*		*		*	
Criteria: Traffic	*		*		**− **0.49	< 0.01	**− **0.70	< 0.01
Criteria: Driving	**− **0.05	0.13			0.14	0.07	0.09	0.13
Criteria: Passenger	*		*		*		*	
Criteria: Stationary in Vehicle	*		*		*		*	
Criteria: Car Parts	*		*		*		0.12	0.06
Criteria: Brand	*		*		0.21	< 0.01	0.19	< 0.01
Criteria: Taxi/Hitching a Ride	**− **0.17	0.06	**− **0.12	0.01	*		*	
Adjusted $r^{2}$	0.03		0.04		0.10		0.15	
Model significance	< 0.01		< 0.01		< 0.01		< 0.01	

^*^Factors with *p *value > 0.15 were excluded

**Table 8:** Regression analysis for sentiment of automobile references (before-after peak car model)

	Alchemy		Joy Watson		Human Analyst 1		Human Analyst 2	
	Coefficients	*p *value	Coefficients	*p *value	Coefficients	*p *value	Coefficients	*p *value
Constant	Not significant		0.36	< 0.01	0.55	< 0.01	0.43	< 0.01
After peak car			**− **0.06	< 0.01	*		*	
Percentage of female artists			*		**− **0.31	< 0.01	*	
Genre: Pop			0 (fixed)		0 (fixed)		0 (fixed)	
Genre: Hip-Hop/Rap			**− **0.06	0.01	*		0.29	< 0.01
Genre: R&B/Soul			*		**− **0.23	0.02	*	
Genre: Rock			**− **0.05	0.10	**− **0.57	< 0.01	**− **0.28	< 0.01
Genre: Country			*		*		*	
Genre: Other			*		*		*	
Criteria: Cars			*		*		*	
Criteria: Traffic			*		**− **0.49	< 0.01	**− **0.70	< 0.01
Criteria: Driving					0.14	0.07	0.09	0.13
Criteria: Passenger			*		*		*	
Criteria: Stationary in Vehicle			*		*		*	
Criteria: Car Parts			*		*		0.12	0.06
Criteria: Brand			*		0.21	< 0.01	0.19	< 0.01
Criteria: Taxi/Hitching a Ride			**− **0.11	0.03	*		*	
Adjusted $r^{2}$			0.04		0.10		0.15	
Model significance			< 0.01		< 0.01		< 0.01	

^*^Factors with *p *value > 0.15 were excluded

## Footnotes

1 Data are presented as 5-year moving averages (i.e. the number of R&B songs in year 1960 in Fig. 1 is the average number of all R&B music from 1956 to 1960) for ease of distinguishing trends from year-on-year random variation.

2 Throughout this paper, we use the term “Popular” music to refer to music in the Top 40, and the term “Pop” to refer to one specific genre of music.

3 https://www.ibm.com/watson/services/alchemy-language-migration/

4 https://www.ibm.com/cloud/watson-tone-analyzer

5 In the interest of time efficiency, we sampled 10 songs from each year (a 25% sample) for purposes of analyzing whole-song sentiment, rather than perform this analysis for all 2400 songs.

6 Examples of a song whose lyrics were rated differently by the human analysts and the algorithms include:

1. Ed Sheeran’s *Photograph* (2014). Both human analysts judged the lyrics to be negative, as it describes a stressful long-distance relationship. However, the algorithms determined its lyrics to be positive, which may be due to the frequency of relatively positive words/phrases in the song (e.g. “love”, “heal”, “never broken”). Similarly, The Ray’s *Silhouettes* (1957), Andy Williams’ *Butterfly* (1957) were judged to be negative by human analysts but positive by algorithms.
2. The Door’s *Light my fire* (1967) was judged to be positive by both human analysts and negative by the algorithms. It describes a male’s wish to accelerate the relationship between himself and a female. There are many negative words in the lyrics (e.g. untrue, liar, lose), which may be the reason that the algorithms judge the song’s overall sentiment to be negative. Similar, Neil Diamond’s *Sweet Caroline* (1969) and James Taylor’s *You’ve got a friend* (1971) were judged to be positive by human analysts but negative by algorithms.

7 Table 5 used year as an integer-denominated independent variable. We also tested two other models that treated year as 10-year bands or before-after peak car (see “Appendix 3”). In general, the three sets of models provide similar outputs.
